# The tired foot. The effects of physical activity, energy expenditure and sleep on the diabetic foot population

**DOI:** 10.1186/1757-1146-8-S2-O35

**Published:** 2015-09-22

**Authors:** Helen Sheahan, Kimberley Canning, Nishka Refausse, Greg Jorgensen, Betty Mulder, Ewan M Kinnear, James Walsh, Peter A Lazzarini

**Affiliations:** 1 Department of Podiatry, Metro North Hospital & Health Service, Queensland Health, Brisbane, Queensland, 4032, Australia; 2 Community Diabetes Service, Metro North Hospital & Health Service, Queensland Health, Brisbane, Queensland, 4032, Australia; 3 Allied Health Research Collaborative, Metro North Hospital & Health Service, Queensland Health, Brisbane, Queensland, 4032, Australia; 4 School of Clinical Sciences, Queensland University of Technology, Brisbane, Queensland, 4059, Australia; 5 Department of Sleep Science, The Prince Charles Hospital, Queensland Health, Brisbane, Queensland, 4032, Australia; 6 Department of Physiotherapy, The Prince Charles Hospital, Queensland Health, Brisbane, Queensland, 4032, Australia

**Keywords:** Diabetes, foot ulcers, sleep, energy expenditure, physical activity, neuropathy

## Background

The most common pathway to development of diabetes foot ulcers is repetitive daily activity stress on the plantar surface of the neuropathic foot. Studies suggest an association between different diabetic foot complications and physical activity. However, to the best of the authors knowledge the steps/day and sleep patterns of people with diabetic foot ulcers has yet to be investigated. This observational study aims to investigate the physical activity and sleep patterns of three groups of adults with type 2 diabetes and different foot complications

## Methods

Participants with type 2 diabetes were recruited into three groups: 1. those with no reported foot complications (DNIL), 2. those with diagnosis of neuropathy (DPN) and 3. those with a neuropathic ulcer (DFU). Exclusion criteria included peripheral arterial disease and mobility aid use. Participants wore a SenseWear Pro 3 Armband continuously for 7 days and completed an Epworth Sleepiness Scale. The Armband is a validated automated measure of activity (walking steps, average Metabolic Equivalent Task (MET), physical activity (>3 METs) duration), energy expenditure(kJ) (total and physical activity (>3 METs)) and sleep (duration). Data on age, sex, BMI, diabetes duration and HbA1c were also collected.

## Results

Sixty-Six (14 DNIL, 22 DPN and 30 DFU's participants were recruited; 71% males, mean age 61(±12) years, diabetes duration 13(±9) years, HbA1c 8.3(±2.8), BMI 32.6(±5.9), average METs 1.2(0.2). Significant differences were reported in mean(SD) steps/day (5,859(±2,381) in DNIL; 5,007(±3,349) in DPN and 3,271(±2,417) in DFU's and daily energy expenditure (10,868(±1,307)kJ in DNIL; 11,060(±1,916)kJ in DPN and 13,006(± 3,559) in DFU's(p <0.05). No significant differences were reported for average METs, physical activity duration or energy expenditure, sleep time or Epworth score (p>0.1).

## Conclusions

Preliminary findings suggest people with diabetes are sedentary. Results indicate that patients with a diabetic foot ulcer work significantly less than those with neuropathy or nil complications and use significantly more energy to do so. Sleep Parameters showed no differences. Recruitment is still on going.

